# Supplementary material for: Programmable viscoelasticity in protein-RNA condensates with disordered sticker-spacer polypeptides
Source: Nat Commun. 2021 Nov 16;12:6620. doi: 10.1038/s41467-021-26733-7 (PMC8595643; doi:10.1038/s41467-021-26733-7)
Supplement: Supplementary file 1 — Supplementary Information [file 41467_2021_26733_MOESM1_ESM.pdf]

Supplementary Information for

Programmable Viscoelasticity in Protein-RNA Condensates with Disordered Sticker-Spacer Polypeptides

Ibraheem Alshareedah[1], Mahdi Muhammad Moosa[1], Matthew Pham[2], Davit A. Potoyan[2]*, Priya R. Banerjee[1]*

[1]Department of Physics, University at Buffalo, Buffalo NY 14260, USA

[2]Department of Chemistry, Iowa State University, Ames IA 50011, USA

*All correspondence could be addressed to:

Priya R. Banerjee (email: prbanerj@buffalo.edu)

Davit A. Potoyan (email: potoyan@iastate.edu)

Supplementary Table 1. Amino acid sequences of the peptides used in this study.

Peptide name	Sequence
$[RGRGG]_5$ and RG0Y	RGRGG RGRGG RGRGG RGRGG RGRGGC
$[KGKGG]_5$	KGKGG KGKGG KGKGG KGKGG KGKGGC
$[RGFGG]_5$	RGFGG RGFGG RGFGG RGFGG RGFGGC
$[RGYGG]_5$ and RG5Y	RGYGG RGYGG RGYGG RGYGG RGYGGC
$[RGSGG]_5$	RGSGG RGSGG RGSGG RGSGG RGSGGC
$[RGPGG]_5$	RGPGG RGPGG RGPGG RGPGG RGPGGC
$[RPRPP]_5$	RPRPP RPRPP RPRPP RPRPP RPRPPC
$[RARAA]_5$	RARAA RARAA RARAA RARAA RARAAC
$[RQRQQ]_5$	RQRQQ RQRQQ RQRQQ RQRQQ RQRQQC
$[RLRLL]_5$	RLRLL RLRLL RLRLL RLRLL RLRLLC
RG1Y	RGRGG RGRGG RGYGG RGRGG RGRGGC
RG3Y	RGRGG RGYGG RGYGG RGYGG RGRGGC
TAF15-RGG box	RGYGGDRGYGGDRGYGGDRGYGGDRGYGGDC
EWS-RGG box	RGRGGPGGMRGGRGGLMDRGGPGGMFRGGRGGC
FUS-RGG box	RRGGRGGYDRGGYRGRGGDRGGFRGGRGGGDRGC

Supplementary Table 2. Viscosity and terminal relaxation time of the various peptide-rU40 condensates tested in this study.

Peptide-RNA	Viscosity (Pa.s)	Terminal relaxation time (ms)
$[RGRGG]_5$ and RG0Y	5 ± 1	60 ± 10
$[KGKGG]_5$	0.26 ± 0.06	N/A
$[RGFGG]_5$	10 ± 2	120 ± 20
$[RGYGG]_5$ and RG5Y	40 ± 10	800 ± 400
$[RGSGG]_5$	0.40 ± 0.06	N/A
$[RGPGG]_5$	0.19 ± 0.09	N/A
$[RPRPP]_5$	1.3 ± 0.2	N/A
$[RARAA]_5$	6 ± 2	50 ± 20
$[RQRQQ]_5$	2.7 ± 0.3	22 ± 5
$[RLRLL]_5$	N/A	N/A
RG1Y	8 ± 2	100 ± 20
RG3Y	13 ± 1	200 ± 70

Supplementary Figures

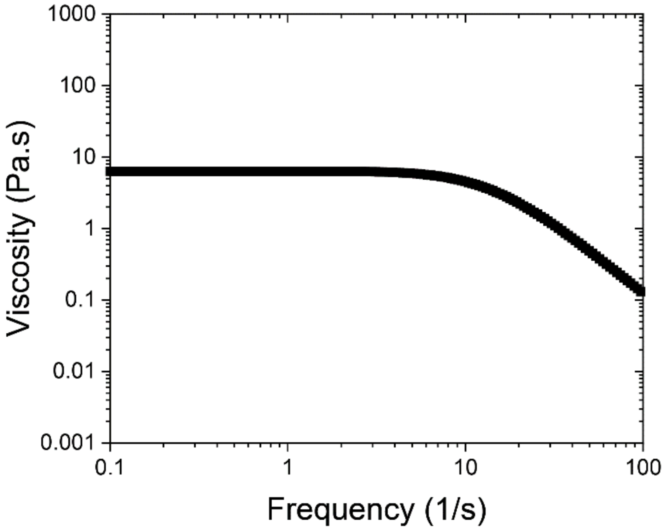

Supplementary Fig. 1. The viscosity of $[RGRGG]_5$-rU40 condensates as a function of frequency as obtained from the pMOT experiments.

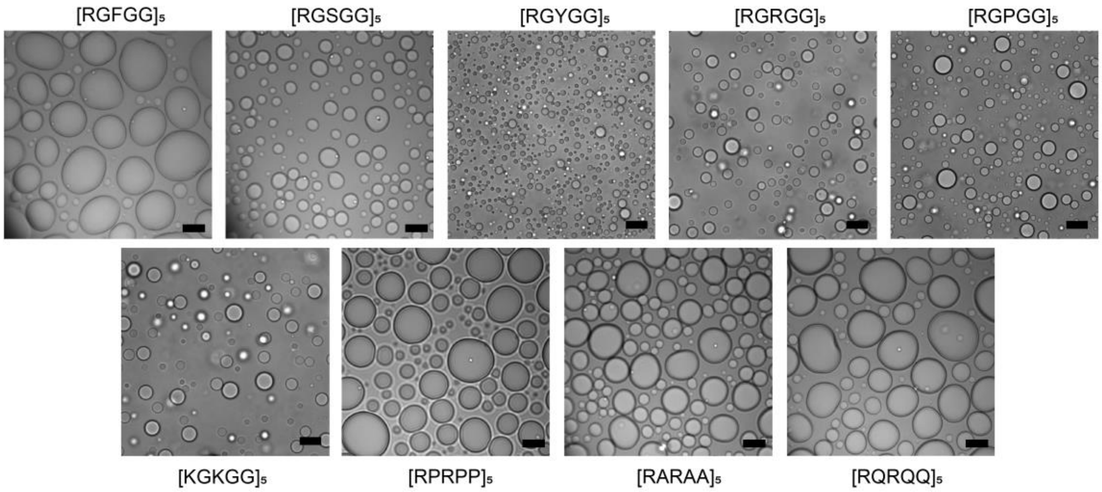

Supplementary Fig. 2. Brightfield images showing spherical condensates formed by various peptides when mixed with rU40 RNA. Scale bars represent 10 µm.

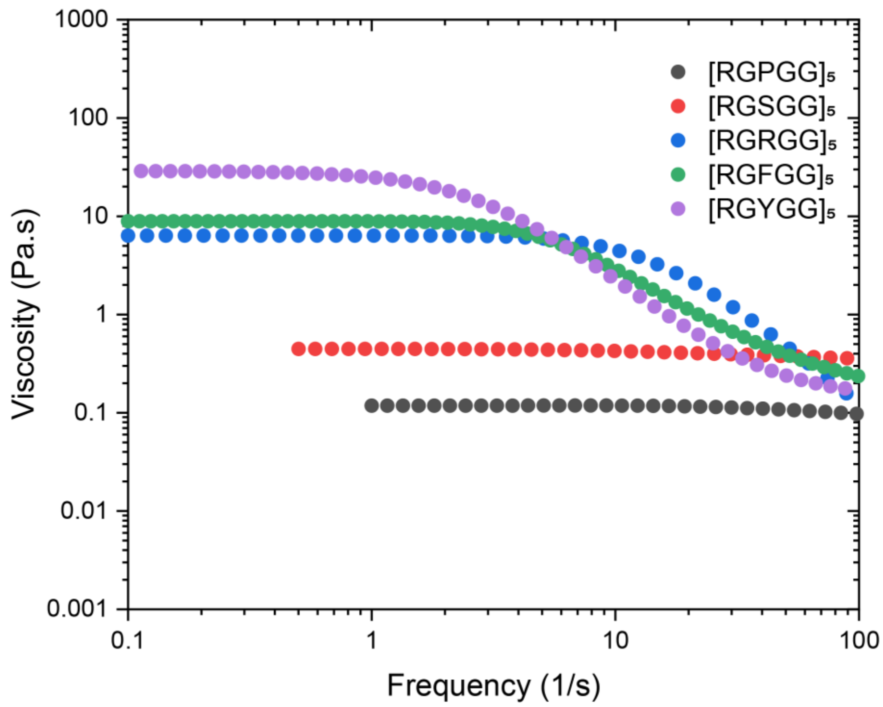

Supplementary Fig. 3. The viscosity of RGXGG-rU40 condensates as a function of frequency as obtained from pMOT experiments. X is a variable amino acid that is set to P, S, R, F, or Y.

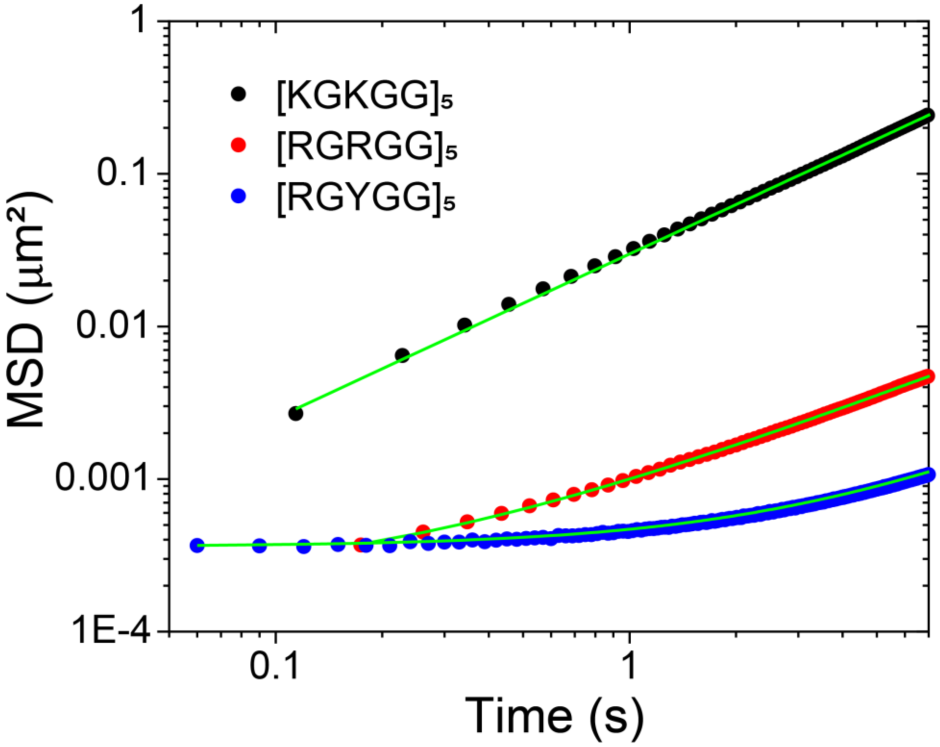

Supplementary Fig. 4. The mean square displacement (MSD) of 200 nm polystyrene particles within condensates formed by rU40 and $[RGPGG]_5$ (black), $[RGRGG]_5$ (red), and $[RGYGG]_5$ (blue). Green lines are fits to the data using $MSD = 4Dt + b$ for $[RGPGG]_5$ and $MSD = d^2(1 + \tau/\tau_c\ )$ for $[RGYGG]_5$ and $[RGRGG]_5$ data[1].

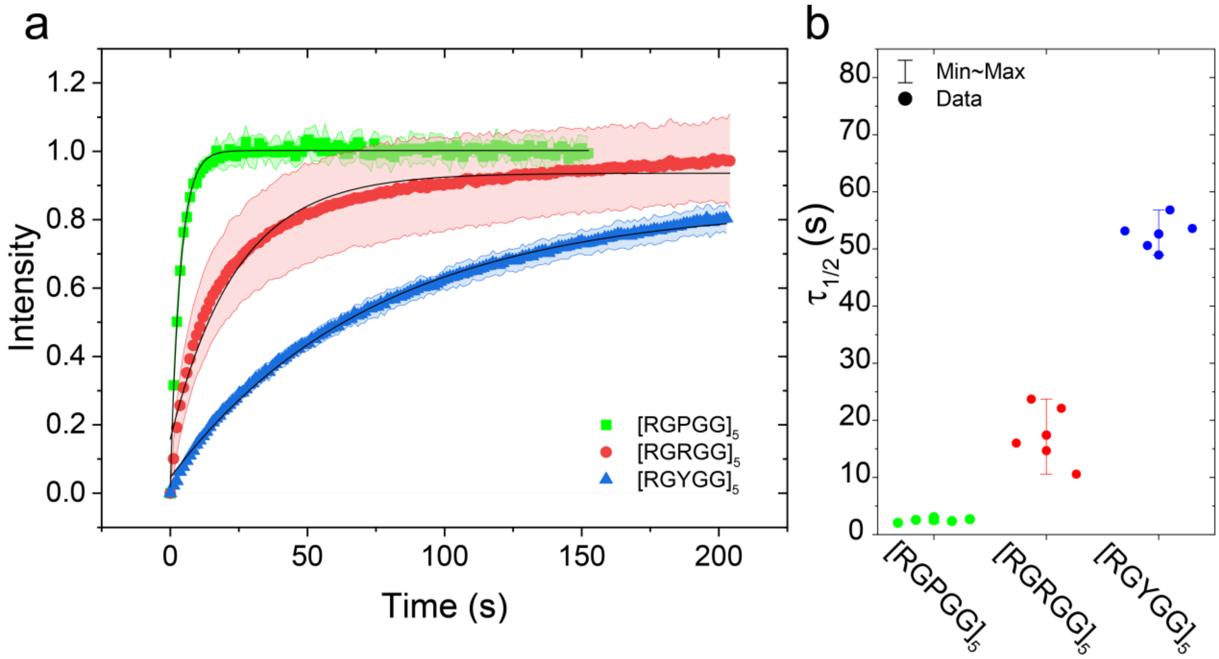

Supplementary Fig. 5. **(a)** FRAP intensity-time traces of an RNA probe (rU10-FAM) in condensates formed by rU40 RNA and $[RGPGG]_5$ (green), $[RGRGG]_5$ (red), and $[RGYGG]_5$ (blue). Solid symbols are the average of five FRAP curves for each peptide-RNA combination. Error bars in the curves represent one standard deviation (±1 s.d.). Solid black lines are fits using $I(t) = A(1 - \exp(-t/\tau)$. **(b)** The recovery half-time ($\tau_{1/2} = \tau \ln 2$) of the same condensates as extracted from the data by fitting individual FRAP recovery traces. Error bars represent the range of the data. For all the peptide-RNA mixtures tested, the sample size is n=5 condensates.

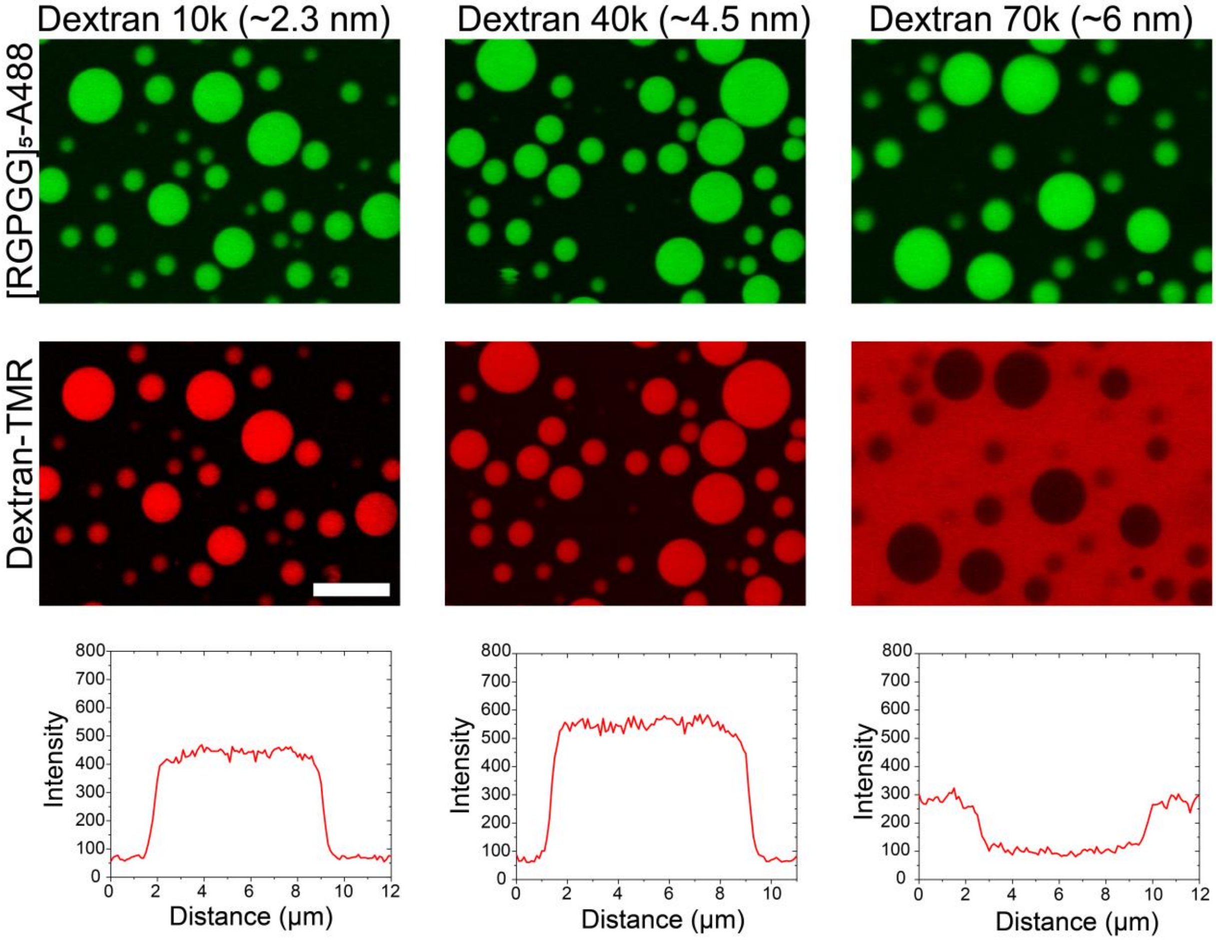

Supplementary Fig. 6. Mesh-size determination experiment using TMR-labeled Dextran molecules with variable molecular weights for $[RGPGG]_5$-rU40 condensates. The upper panel shows the condensates as visualized by Alexa488-tagged $[RGPGG]_5$ (1% labeling ratio). The middle panel shows the partition behavior of Dextran molecules within peptide-RNA condensates. The scale bar represents 10 µm. The lower panel shows corresponding intensity profiles for Dextran molecules. The numbers in brackets indicate the estimated hydrodynamic radius of the Dextran molecules in aqueous solutions[2].

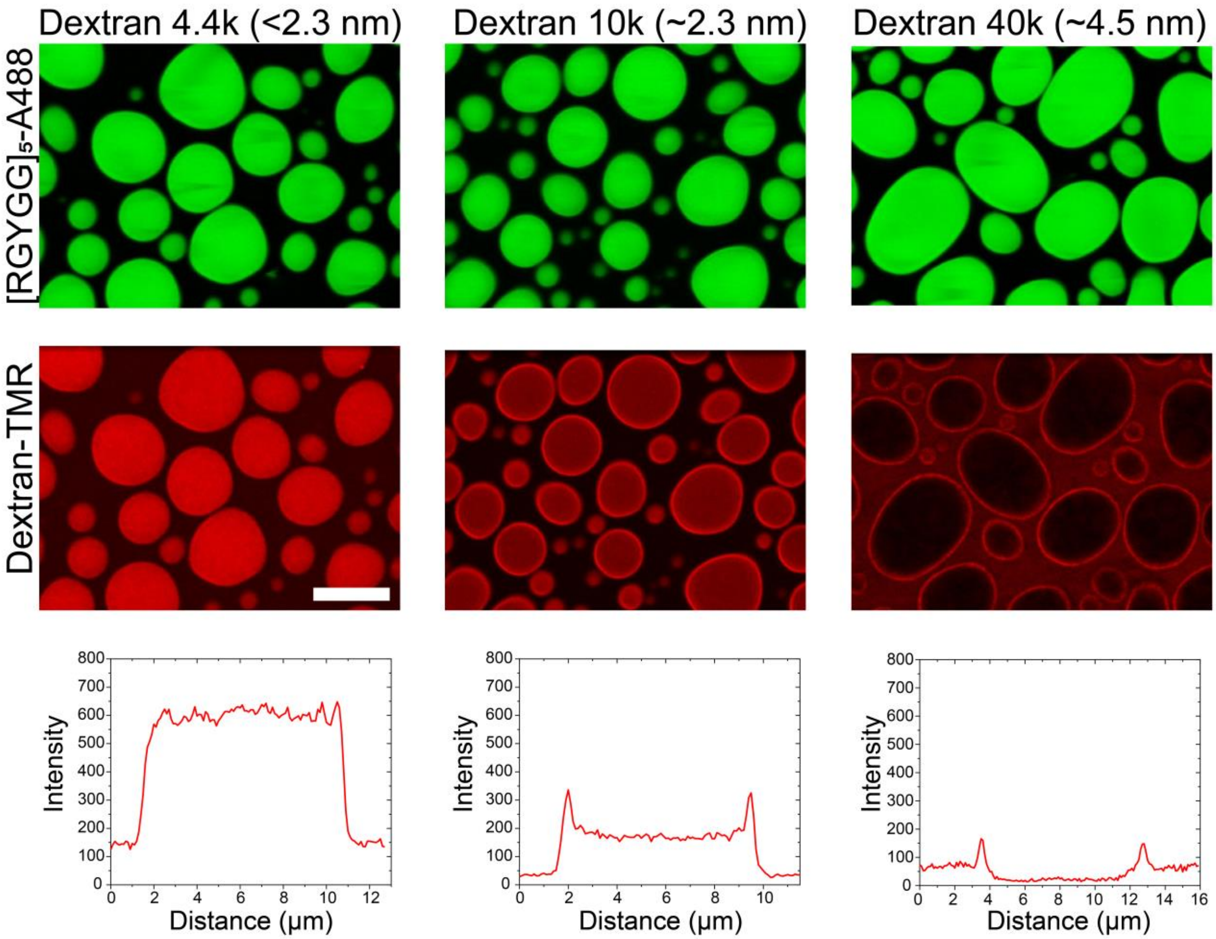

Supplementary Fig. 7. Mesh-size determination experiment using TMR-labeled Dextran molecules with variable molecular weights for $[RGYGG]_5$-rU40 condensates. The upper panel shows the condensates as visualized by Alexa488-tagged $[RGYGG]_5$ (1% labeling ratio). The middle panel shows the partition behavior of Dextran molecules within peptide-RNA condensates. The scale bar represents 10 µm. The lower panel shows corresponding intensity profiles for Dextran molecules. The numbers in brackets indicate the estimated hydrodynamic radius of the Dextran molecules in aqueous solutions[2].

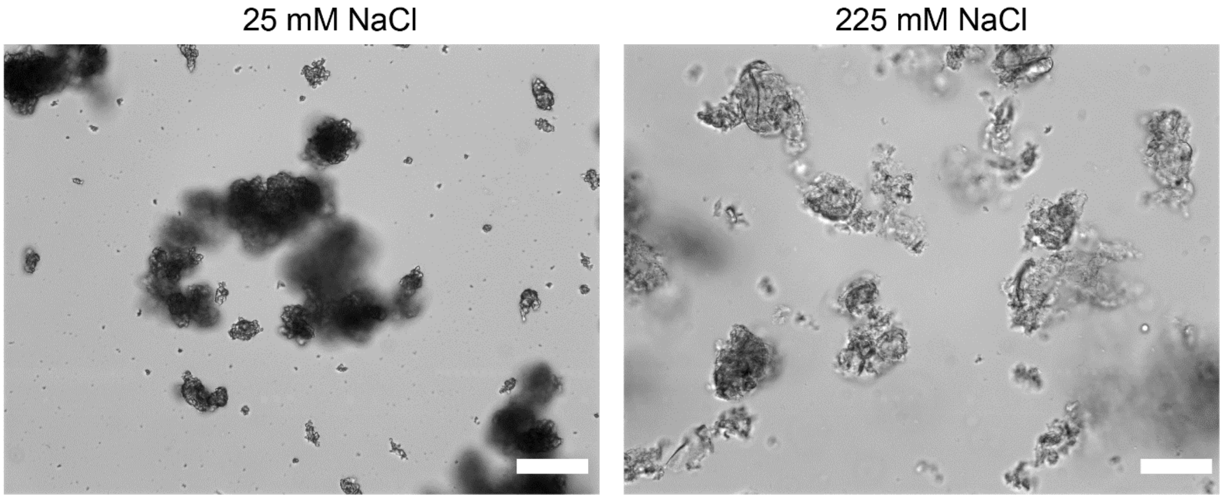

Supplementary Fig. 8. Bright-field images of arrested condensate networks formed by $[RLRLL]_5$ and rU40 RNA at two different salt conditions in 25 mM Tris-HCl buffer (pH 7.5) and 20 mM DTT. Scale bars represent 20 µm.

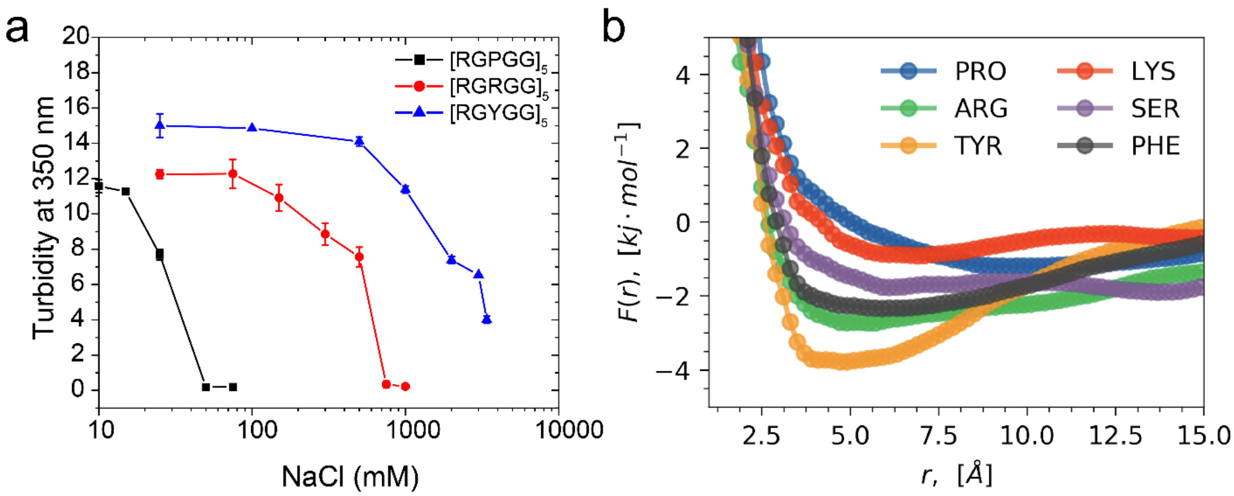

Supplementary Fig. 9. (a) Solution turbidity at 350 nm wavelength for peptide-rU40 condensate as a function of salt concentration in 25 mM Tris-HCl buffer (pH 7.5). Data are shown for $[RGPGG]_5$, $[RGRGG]_5$, and $[RGYGG]_5$ condensates with rU40 RNA. Each sample was measured twice (n=2) and the measurements were averaged. Error bars represent the range of the measurements. **(b)** Free energy profiles of residue-rU attraction from model (GXG)-rU3 all-atom constant temperature simulations. X is set to Pro, Arg, Tyr, Lys, Ser, or Phe.

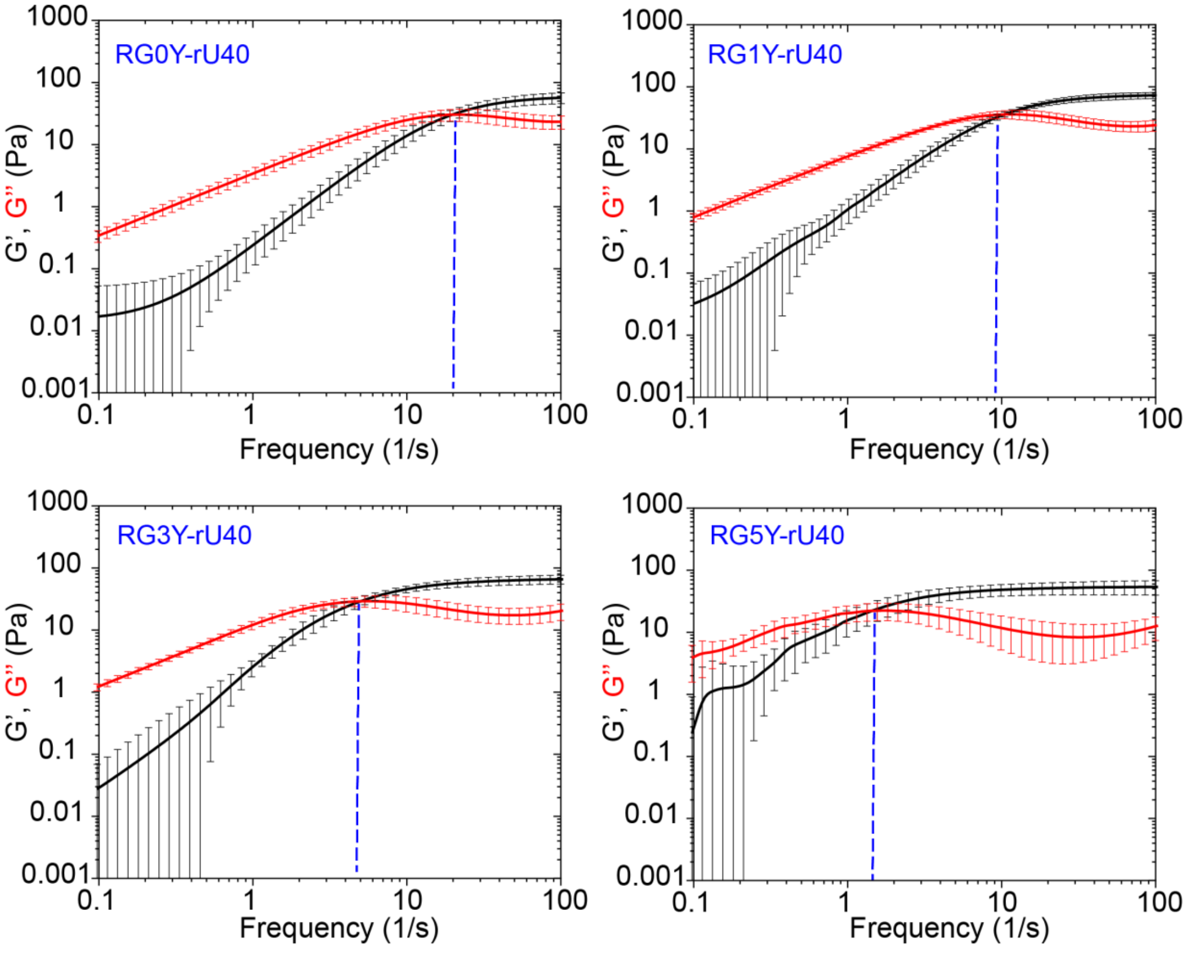

Supplementary Fig. 10. The average frequency-dependent viscoelastic moduli as obtained from pMOT experiments for condensates formed by RGnY and RNA (see Supplementary Table 1 for peptide sequences). Here n is the number of R-to-Y mutations in the 2nd arginine of RGRGG-repeat peptide. $[RG\underline{R}GG]_5$ corresponds to RG0Y and RG5Y corresponds to $[RG\underline{Y}GG]_5$. The blue dashed lines indicate the crossover frequency. Error bars represent ±1 s.d. Sample size is n=(17,20,10,16) for (RG0Y, RG1Y, RG3Y, RG5Y), respectively.

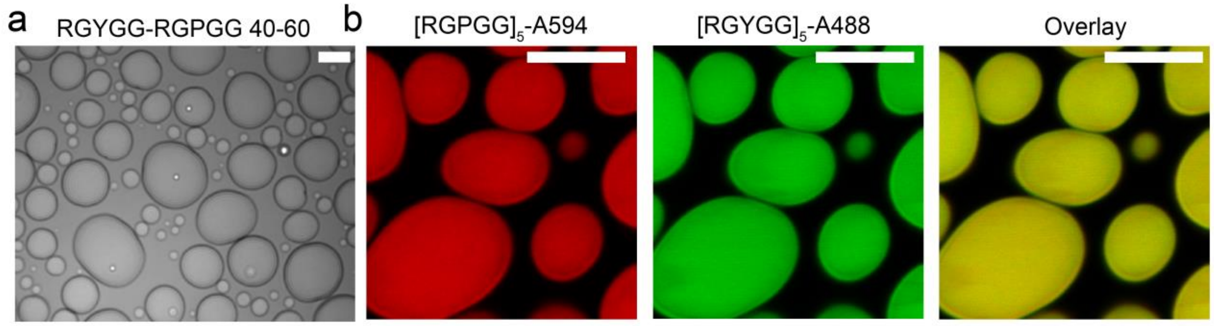

Supplementary Fig. 11. **(a)** Brightfield image of condensates formed by $[RGYGG]_5$, $[RGPGG]_5$, and rU40 RNA. The total peptide concentration is 5 mg/ml (with 40% $[RGYGG]_5$ and 60% $[RGYGG]_5$). The RNA concentration is 2.5 mg/ml. The image shows that these droplets are homogenous and well-mixed. The sample also contained 1 µm beads for pMOT experiments. **(b)** Multi-color fluorescence images of condensates formed by mixtures of $[RGYGG]_5$ and $[RGPGG]_5$ in the presence of rU40 RNA. In this sample, the RGPGG-to-RGYGG ratio is 50:50. ~ 1% of Alexa-fluorophore-labeled peptides are used for imaging, as indicated. The scale bar is 10 µm.

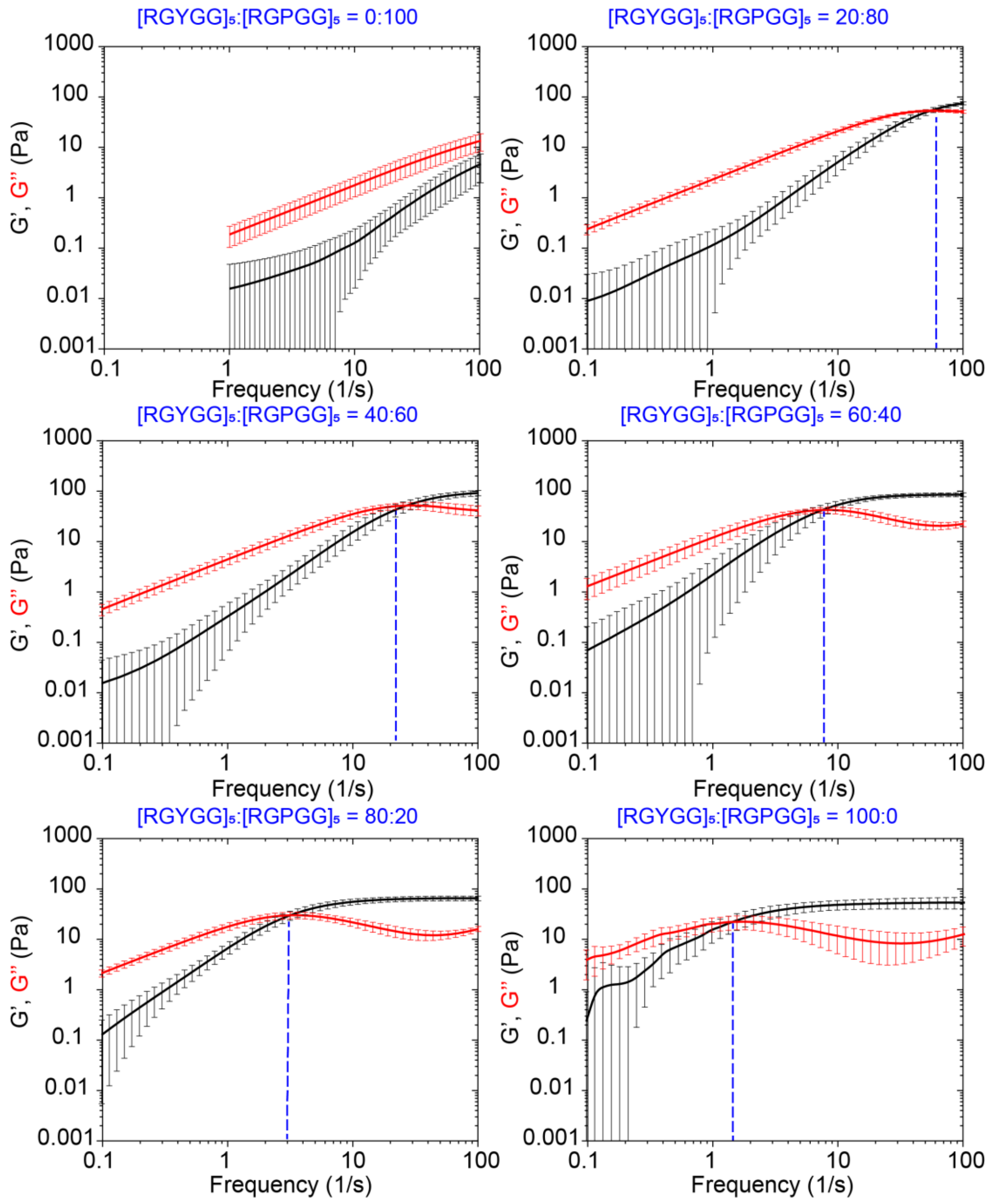

Supplementary Fig. 12. The average frequency-dependent viscoelastic moduli as obtained from pMOT experiments for condensates formed in a ternary mixture of $[RGYGG]_5$, $[RGPGG]_5$, and rU40. In these mixtures, the total peptide concentration is fixed at 5.0 mg/ml, the total rU40 concentration is 2.5 mg/ml. The molar fractions of $[RGYGG]_5$ and $[RGPGG]_5$ are varied while keeping the overall peptide concentration fixed to illustrate the composition-dependent viscoelastic modulation of these condensates. The blue dashed lines indicate the crossover frequency. Error bars represent ±1 s.d. Sample size is n = (29,14,36,18,18,16) for $[RGYGG]_5$ fractions of (0%, 20%, 40%, 60%, 80%, 100%), respectively.

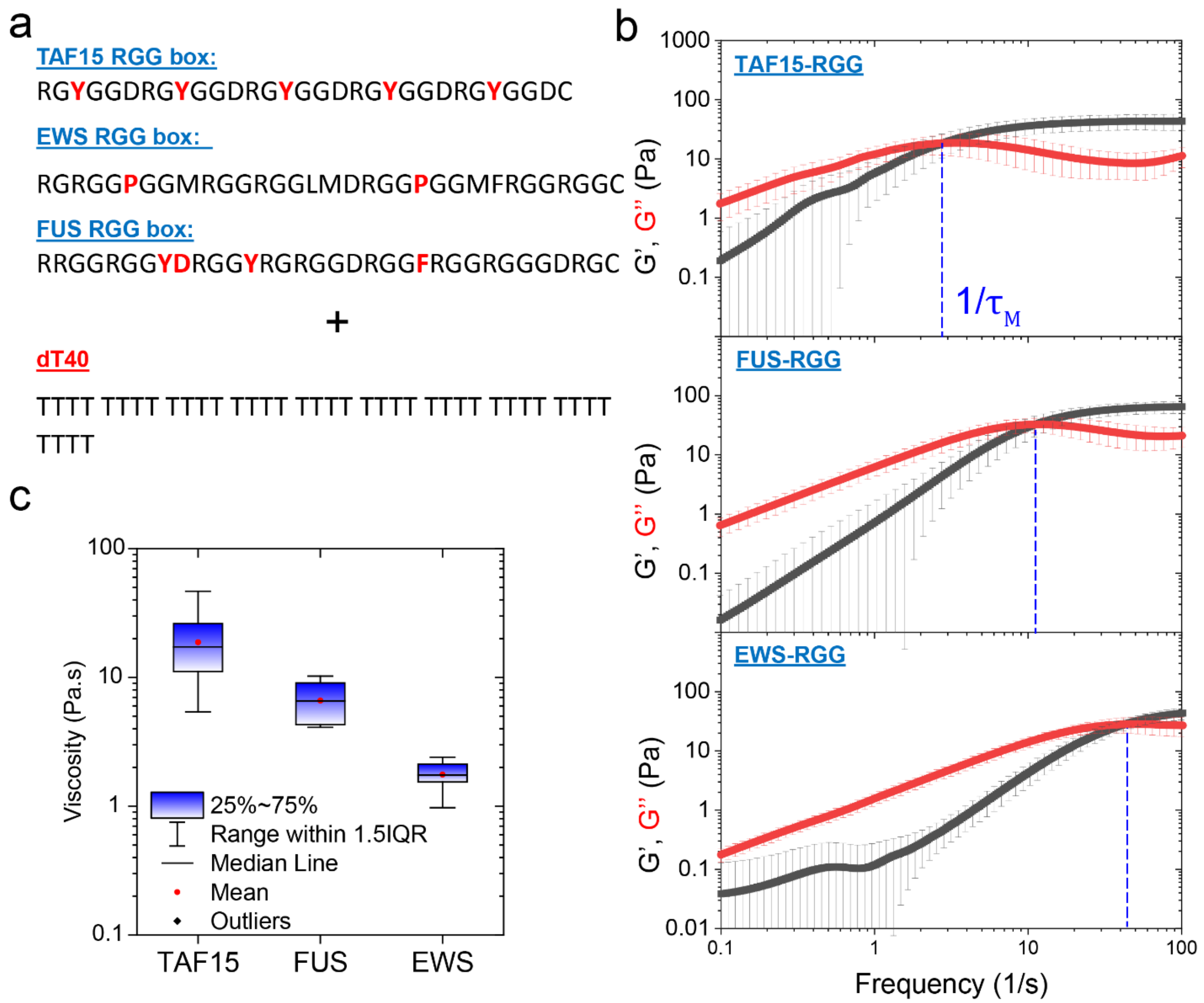

Supplementary Fig. 13. pMOT measurements on the RGG boxes of FET proteins showing distinct viscoelastic properties. **(a)** Sequences of the RGG boxes derived from FUS, EWS, and TAF15. **(b)** The average viscoelastic moduli of condensates formed by TAF15-RGG, FUS-RGG, or EWS-RGG with dT40. The blue dashed line indicates the inverse terminal relaxation time. Error bars represent ±1 s.d. **(c)** Zero-shear viscosity of condensates formed by TAF15-RGG, FUS-RGG, or EWS-RGG with dT40. For **b&c**, the sample size is n = (13,10,20) for (TAF15-RGG, FUS-RGG, EWS-RGG), respectively.

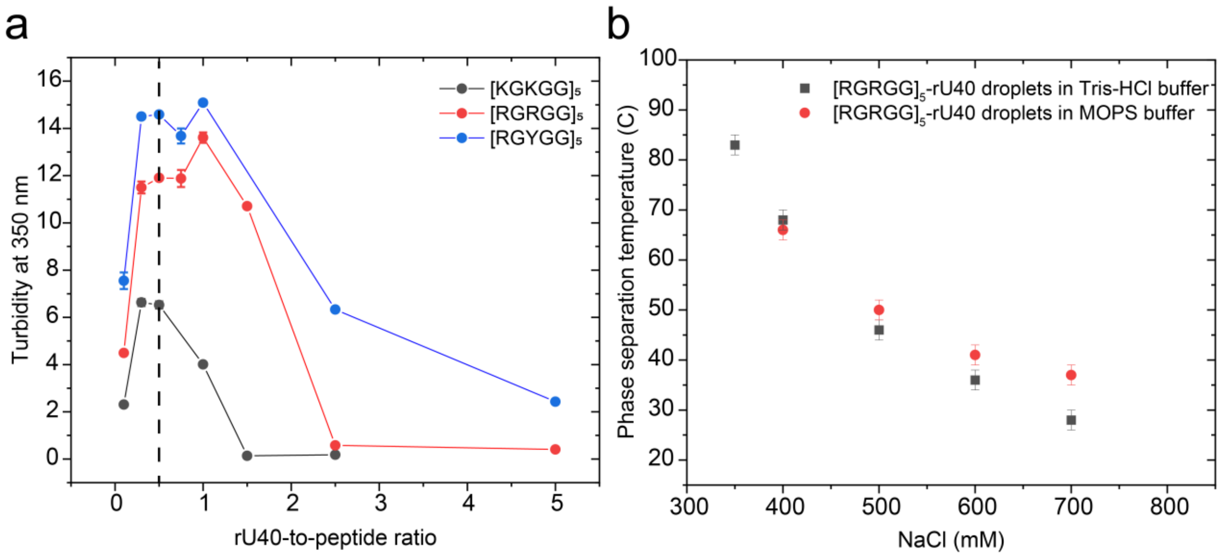

Supplementary Fig. 14. **(a)** Turbidity of peptide-RNA mixtures as a function of RNA (rU40)-to-peptide weight ratio. The data are shown for $[KGKGG]_5$, $[RGRGG]_5$, and $[RGYGG]_5$ mixtures with rU40 RNA. The vertical dashed line represents the chosen ratio for the experiments in this study (RNA-to-peptide ratio of 0.5). Sample size is n=2 measurements, error bars are the range of measurements and the solid symbols are the averages. **(b)** Comparison of phase separation temperature (T_{ph}) for $[RGRGG]_5$-rU40 condensates mixed in a buffer containing 25 mM Tris-HCl (pH 7.4) with variable salt and a buffer containing 25 mM MOPS (pH 7.4) and variable salt concentration. Both buffers also contain 20 mM DTT. This comparison is shown to assess the effect of temperature on the pH of the Tris buffer that might alter the phase separation behavior. The Tris buffer shows a similar trend of phase separation temperatures as compared to the MOPS buffer which has lesser pH sensitivity to temperature[3]. Solid symbols represent the measured phase separation temperature (T_{ph}). Error bars represent the smallest temperature variation (2 ^{o}C) during a phase transition event. Random points were replicated twice successfully.

Supplementary Note 1

Data analysis:

The analysis of pMOT experiments is summarized in a flow chart and shown in Supplementary Figure 15. The output of a pMOT experiment is a trajectory of the bead (Supplementary Fig. 15a) constrained by the harmonic potential of an optical trap within a peptide-RNA condensate. Each trajectory was analyzed in two steps: (i) calibration of the optical tweezer, and (ii) extraction of the condensate viscoelastic moduli. Both steps are done on the same trajectory.

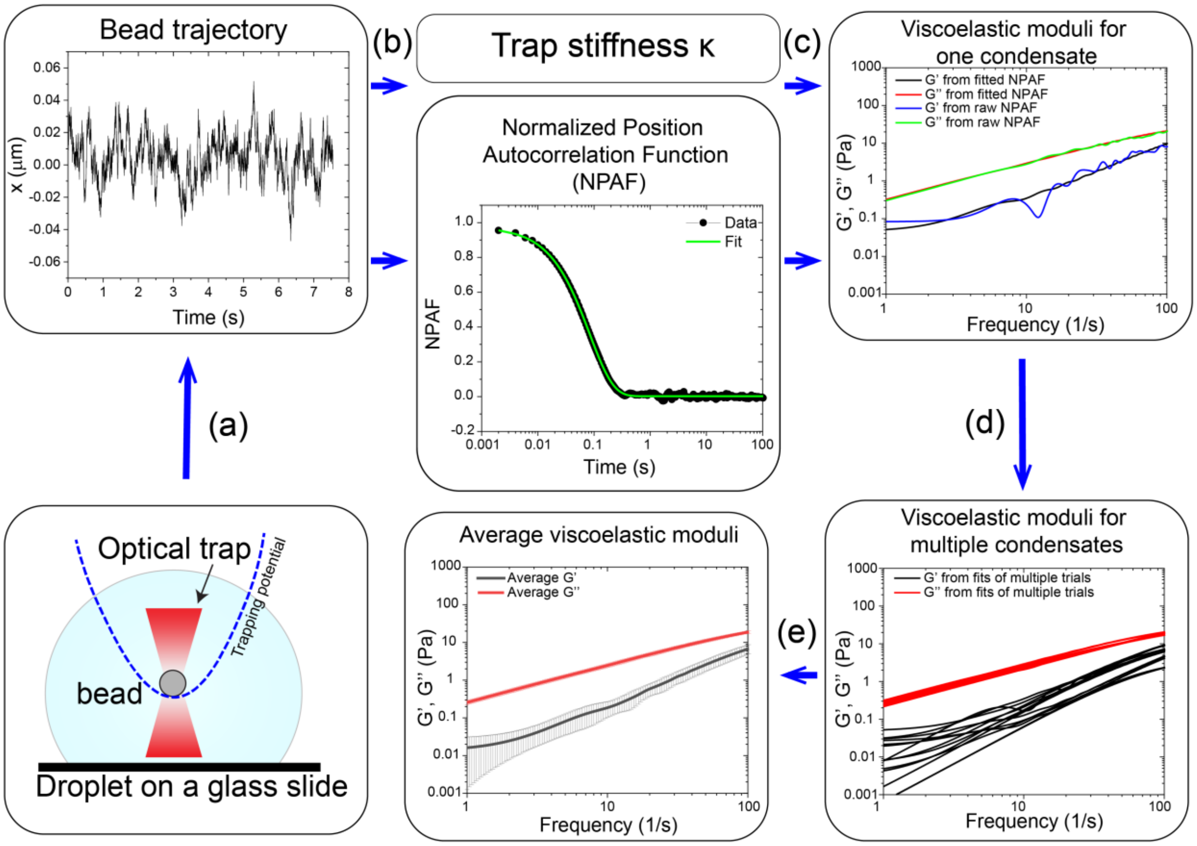

Supplementary Fig. 15. Flow chart showing pMOT data analysis procedure to obtain the viscoelastic moduli of phase-separated condensates. **(a-e)** various steps of the procedure are described in the text of SI Note-1.

When a bead is diffusing within the harmonic potential of an optical trap, the motion of the bead is primarily mediated by the thermal fluctuations of the medium. According to the equipartition theorem, the variance of the position of the bead in one direction (X or Y) is given by the following equation[4-8]

$$\frac{1}{2}\kappa\langle x^2\rangle = \frac{1}{2}k_BT \qquad (1)$$

Where κ is the trap stiffness, k_B is the Boltzmann constant, and T is the temperature. For each recorded trajectory, the variance in both the X and Y coordinates was calculated from the trajectory. The centroid position accuracy was measured to be 1.5 nm (Supplementary Fig. 16). The temperature of the stage was manually measured using a thermocouple and found to be 24 ± 0.5 ^{o}C. We ensured only using trajectories for which the variance in the X and Y directions were identical, indicating that the trapping potential is circular in the XY plane and therefore, the estimation of the trap stiffness from the harmonic form is valid. The values for the trap stiffness in the X and Y directions were compared and found to be consistent with each other for each measurement. This calibration method is one of the few methods that do not require a priori knowledge of the viscosity of the medium[4].

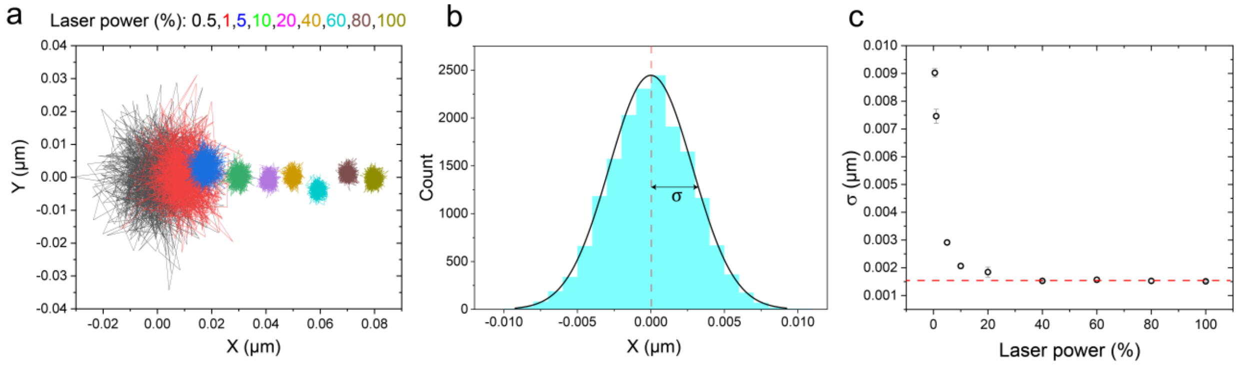

Supplementary Fig. 16. (a) Trajectories of a bead trapped in water at different laser trapping powers (100% laser is ~1 mW). **(b)** Displacement distribution of the bead from the center of the trap. Data is shown for trapping power of 5 %. σ is the standard deviation of the fluctuations. **(c)** The standard deviation ($\sigma = \sqrt{\text{variance}}$) of the trapped bead position fluctuations under different laser trapping power values. The values of σ saturate at 1.5 nm, which is taken as the centroid positioning accuracy. For each laser power, two measurements were collected and averaged (n=2). Error bars represent the range of measurements.

For a particle undergoing Brownian motion in a fluid and constrained by an optical trap, the particle's motion can be described by a generalized Langevin equation similar to the one described by Mason and Weitz[9] with an added term for the optical trap[4]

$$m\vec{a}(t) = \overrightarrow{f_R}(t) - \int_0^t \zeta(t-\tau)\vec{v}(\tau)\, d\tau - \kappa\vec{r}(t) \tag{2}$$

Where m is the mass of the particle and $\vec{a}$ is the acceleration. The first term $\overrightarrow{f_R}(t)$ represents a white noise function to model the thermal fluctuations. The second term involves a memory function $\zeta(t-\tau)$ that represents damping forces exerted by the fluid on the particle [$\vec{v}(\tau)$ is the particle velocity], and the third term is the optical trap force. In one dimension, equation (2) can be rewritten as a differential equation in terms of the position of the particle[4,10,11]

$$m\ddot{r}(t) = f_R(t) - \int_0^t \zeta(t-\tau)\dot{r}(\tau)\, d\tau - \kappa r(t) \tag{3}$$

Where the dot accent represents a time derivative. To solve this equation, the method of Laplace transforms is used. The solution in the Laplace space is given by[4,10,11]

$$\tilde{\zeta}(s) = \kappa\frac{\tilde{A}(s)}{1-s\tilde{A}(s)} - ms \tag{4}$$

Where $A(s)$ is the normalized position autocorrelation function in the Laplace space. At the same time, the memory function was evaluated by Mason and Weitz in relation to the viscosity of the medium under equilibrium conditions as[9]

$$\tilde{\zeta}(s) = 6\pi a \tilde{\eta}(s) \tag{5}$$

Hence, combining the two equations (4 and 5) gives a relation between the viscosity and the autocorrelation function in the Laplace space[4]

$$\tilde{\eta}(s) = \frac{\kappa}{6\pi a}\left(\frac{\tilde{A}(s)}{1 - s\tilde{A}(s)} - \frac{ms}{\kappa}\right) \tag{6}$$

The viscosity in the Laplace space can be transformed into the complex modulus in the Fourier space using[4]

$$G^*(\omega) = s\tilde{\eta}(s)|_{s=i\omega} \tag{7}$$

Thus, the final expression for the complex modulus in terms of the particle normalized position autocorrelation function is expressed as[4,11,12]

$$G^*(\omega) = \frac{\kappa}{6\pi a}\left(\frac{i\omega\hat{A}(\omega)}{1 - i\omega\hat{A}(\omega)} + \frac{m\omega^2}{\kappa}\right) \tag{8}$$

Where κ is the stiffness of the trap and a is the particle radius. The second term in the bracket is negligible except for very high frequencies $\omega \sim 1\,MHz$. Therefore, we used this equation (neglecting the second term) to obtain the complex shear modulus of the material[4,11,12]

$$G^*(\omega) = G'(\omega) + i\,G''(\omega) = \frac{\kappa}{6\pi a}\left(\frac{i\omega\hat{A}(\omega)}{1 - i\omega\hat{A}(\omega)}\right) \tag{9}$$

Equation (9) was implemented in a custom-built python script and used to extract the elastic modulus $G'(\omega)$ and the viscous modulus $G''(\omega)$ of the condensate. The frequency-dependent viscosity $\eta(\omega)$ was calculated using[11]

$$\eta(\omega) = \frac{G''(\omega)}{\omega} \tag{10}$$

The zero-shear viscosity was obtained from the limit of $\eta(\omega)$ at low frequencies. We calculate the autocorrelation function $A(\tau)$ from a trajectory of a bead within a condensate in one coordinate (X or Y, Supplementary Fig. 15b). This was done using the *multipletau.autocorrelate()* function from the multipletau python library[13] (v0.1.9). The autocorrelation function is fitted with a multi-exponential function to reduce the measurement noise (Supplementary Figs. 15b, 22, and 23)

$$A(\tau) = A_1 \exp\left(-\frac{\tau}{b_1}\right)^{\alpha_1} + A_2 \exp\left(-\frac{\tau}{b_2}\right)^{\alpha_2} + A_3 \exp\left(-\frac{\tau}{b_3}\right)^{\alpha_3} + A_4 \tag{11}$$

Next, we perform a numerical Fourier transform on $A(\tau)$ to obtain $A(\omega)$. Following the procedure described by Evans et al.[14], for a function $g(t)$ with discrete points (t_k, g_k) and boundary conditions $g(0) = 1$ and $\dot{g}(t) \to 0$ as $t \to \infty$,

$$-\omega^2\hat{g}(\omega) = i\omega g(0) + \frac{(1 - e^{-i\omega t_1})(g_1 - g(0))}{t_1} + \dot{g}(\infty)e^{-i\omega t_N} + \sum_{k=2}^{N}\left(\frac{g_k - g_{k-1}}{t_k - t_{k-1}}\right)\left(e^{-i\omega t_{k-1}} - e^{-i\omega t_k}\right) \tag{12}$$

This equation was implemented to calculate $\hat{A}(\omega)$ from the fitted $A(\tau)$. Classical numerical Fourier transform methods require the function to have equidistant time points. However, this method of

Fourier transform does not have that requirement[14]. Finally, $A(\omega)$ was substituted in equation (9) to calculate the complex modulus $G^*(\omega) = G'(\omega) + iG''(\omega)$ (Supplementary Figs. 15c, 24, and 25).

For each bead trajectory from each condensate, we extracted $G'(\omega), G''(\omega),$ and η (Supplementary Fig. 15d). The final viscoelastic moduli from multiple condensates were averaged for each frequency ω. Error bars were estimated using the standard deviation (Supplementary Fig. 15e). The presented values for the zero-shear viscosity are the average of at least 10 values extracted from individual condensates' moduli. The terminal relaxation time was obtained by averaging the inverse of the crossover frequency, which was calculated from G' and G'' frequency traces for individual condensates. An example of the condensate-to-condensate variation of the moduli is depicted in supplementary figure 15d. To ensure the correctness of our calculations, we used this method to measure the viscosity of water found it to be 0.93 ± 0.03 mPa.s which is comparable with the reported value in the literature[15] (0.91 mPa.s, Supplementary Fig. 17).

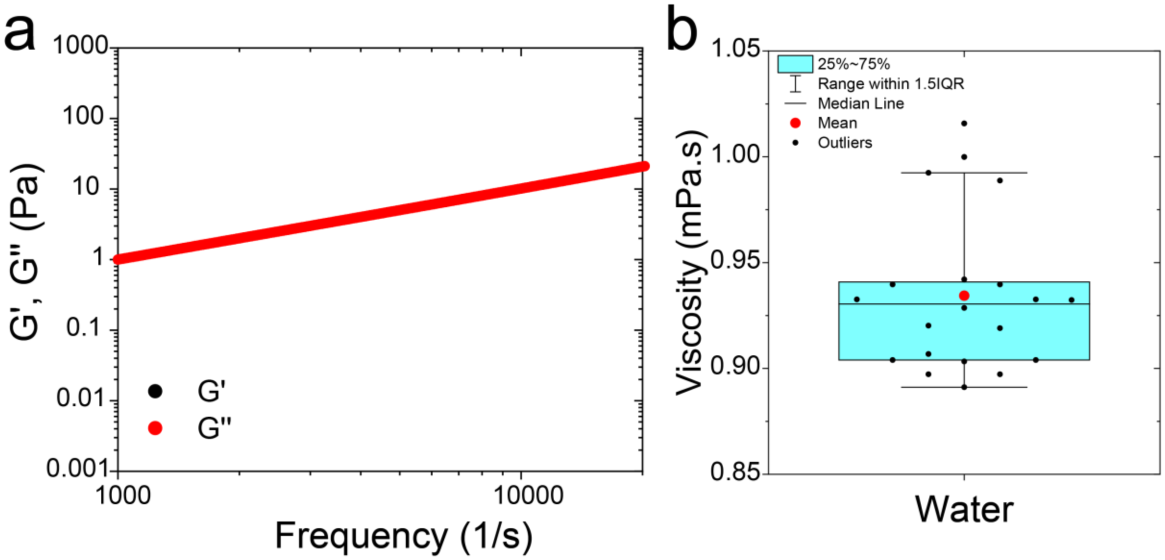

Supplementary Fig. 17. **(a)** Viscoelastic moduli of water using the pMOT assay with 2 µm polystyrene beads. **(b)** The viscosity of water as measured from the pMOT assay (water viscosity is 0.91 mPa.s[15]). Sample size is n=16 measurements.

Bead-condensate interactions:

One of the assumptions of microrheology is that there are no strong interactions between the bead and dense phase. The presence of such interactions alters the extracted rheological response of the material[16]. We have previously probed the effect of bead-condensate interactions on video particle tracking microrheology using $[RGRGG]_5$-dT40 condensates. In that study, we showed that the presence of the carboxylate beads did not alter the overall phase behavior of the peptide-nucleic acid system (Figure S3 in Alshareedah et al.[17]). This means that the beads are not affecting the interactions between the nucleic acids and the peptides significantly. To further confirm that, we performed a bead-halo assay on non-fluorescent carboxylate beads embedded within peptide-RNA condensates. The bead-halo assay is used to probe intermolecular

interactions by coating a bead with a substrate and adding the bait protein[18]. If the protein binds to the substrate it will coat the bead and the bead surface will have an intensity higher than the average bulk intensity[18] (Supplementary Fig. 18a). We used carboxylate functionalized beads which partition positively in peptide-RNA condensates. As our baits, we used $[RGRGG]_5$-Alexa594 and U10-FAM RNA to see if the beads are interacting with the peptide or the RNA inside the condensates. Our experiments show that beads' intensity is less than or equal to the mean intensity of the probes inside the condensates, indicating no significant interactions or adsorption of the biopolymers on the bead surface (Supplementary Fig. 18b). This result is also consistent with a recent study that showed that the MSDs of PEGylated beads and carboxylate beads are identical within condensates formed by polyR10 ($[R]_{10}$) and UTP[19].

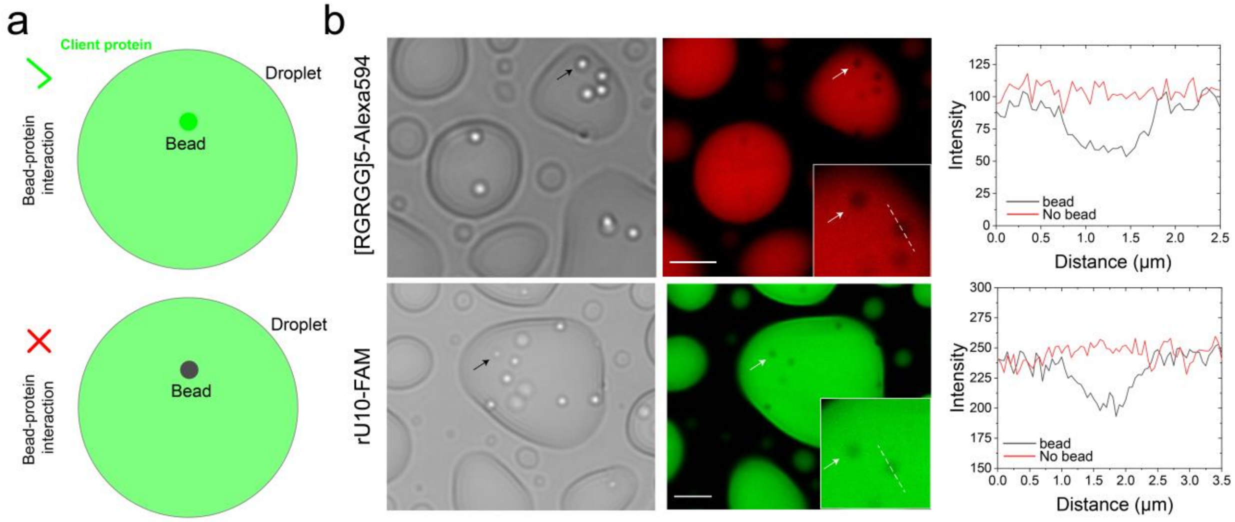

Figure S18. (a) Schematic illustration of the expected outcome of the bead-halo assay in case of the presence and absence of bead-client interactions. **(b)** Bright-field and fluorescent images of carboxylate beads within $[RGRGG]_5$-rU40 condensates and corresponding intensity profiles. (*Top panel*) The recruitment behavior of $[RGRGG]_5$-Alexa594 with the carboxylate beads within peptide-RNA condensates showing that the beads do not recruit or adsorb any peptide molecules on its surface. The intensity profile across the bead (black) shows a dip in intensity due to the absence of any $[RGRGG]_5$-Alexa594 molecules on its surface. The intensity profile across a bead-free region (red) of the droplets shows the average mean intensity. (*Bottom panel*) similar data but with U10-FAM RNA as the client in the bead-halo assay. The scale bars represent 10 µm. These results indicate that the carboxylate beads do not interact significantly with the condensate medium as they do not recruit or concentrate the two components forming the condensates: $[RGRGG]_5$ and RNA.

***Effect of solid and liquid interfaces**:*

For accurate rheological analysis, the effects of nearby solid and liquid interfaces need to be quantified in order to ensure that laser refraction and interfacial fluctuations do not have significant effects on the measurements. First, we confirmed that the proximity of a liquid-liquid interface (~3

μm) does not significantly alter the viscoelastic moduli (Supplementary Fig. 19), even though all of our experiments were performed when the bead is at the center of the condensate (distance from a liquid-liquid interface > 3 μm).

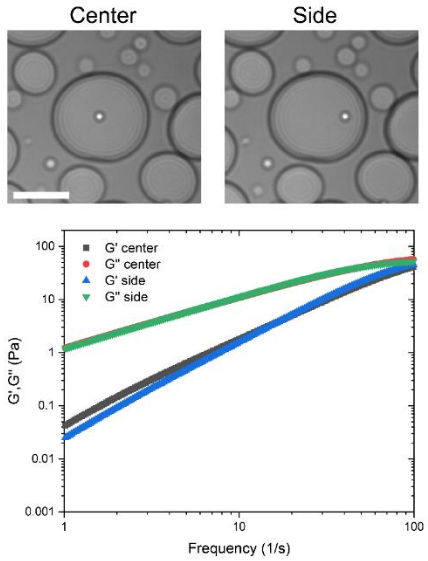

Supplementary Fig. 19. A comparison between the frequency-dependent viscoelastic moduli (G' and G'') as obtained from pMOT experiments at the center of the condensate and at the side of the condensate (~ 3 μm from the condensate interface with the dilute phase). These condensates are formed by mixtures of $[RPRPP]_5$ peptide (5.0 mg/ml) and rU40 RNA (2.5 mg/ml). No significant effects of the proximity to the interface on the viscoelastic moduli were observed under these conditions. The scale bar represents 10 μm.

Next, we probed whether the proximity of the solid glass interface affects the optical trapping and the pMOT measurements due to the reflection and refraction of the trapping laser as well as bead-surface interactions. First, we measured the normalized position autocorrelation function (NPAF) for 1 μm bead trapped at a variable distance from the glass surface h in water using identical trapping power (Supplementary Fig. 20a). We found that there is insignificant variation in the NPAF curves as we change the distance from the surface from 3 μm to 10 μm. The same result was obtained from an identical experiment using a 35% aqueous solution of polyethylene glycol (Supplementary Fig. 20b&c). This indicates that the trajectory of the bead in the X and Y direction 3 μm away from the surface is similar to that when the bead is 10 μm away from the surface.

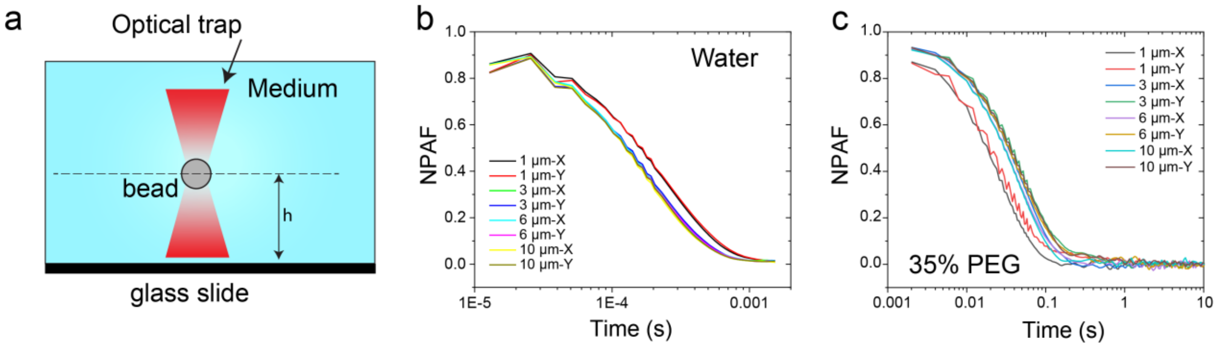

Supplementary Fig. 20. (**a**) A scheme illustrating the trapping of a particle in a continuous medium at a variable distance h from the glass surface. (**b**) Normalized position autocorrelation function (NPAF) of a trapped bead at a variable distance from the surface and at identical laser power within water. (**c**) Normalized position autocorrelation function (NPAF) of a trapped bead at a variable distance from the surface and at identical laser power within a solution of 35 % wt/vol PEG8000.

Next, we trapped a 1 µm bead within a peptide-RNA condensate ($[KGKGG]_5$-rU40) at different distances from the surface h and measured the bead trajectory (Supplementary Fig. 21a). We find that similar to the case of a continuous medium (Supplementary Fig. 20), the NPAF curves were similar for the bead at all the tested surface to bead separations (1µm - 10µm; Supplementary Fig. 21b). Additionally, the displacement probability in the X and Y direction was identical across all the tested surface-to-bead separations (Supplementary Fig. 21c). This indicates that pMOT experiments under these conditions are not affected by the proximity of neither the glass coverslip surface nor the liquid-liquid interface (Supplementary Fig. 19). The Gaussian shape of the displacement probability also indicates that the trapping potential is harmonic[6]. Increasing the laser trapping power led to progressively narrower displacement probability distributions of the trapped bead within the peptide-RNA condensate (Supplementary Fig. 21d). Furthermore, the relation between the measured trap stiffness and the laser power was found to be linear inside the condensate (Supplementary Fig. 21e), which is expected for a harmonic optical trap[7]. Collectively, these data confirm the integrity of the optical trap when present within a peptide-RNA condensate and the validity of the harmonic potential approximation that facilitates our passive microrheology measurements.

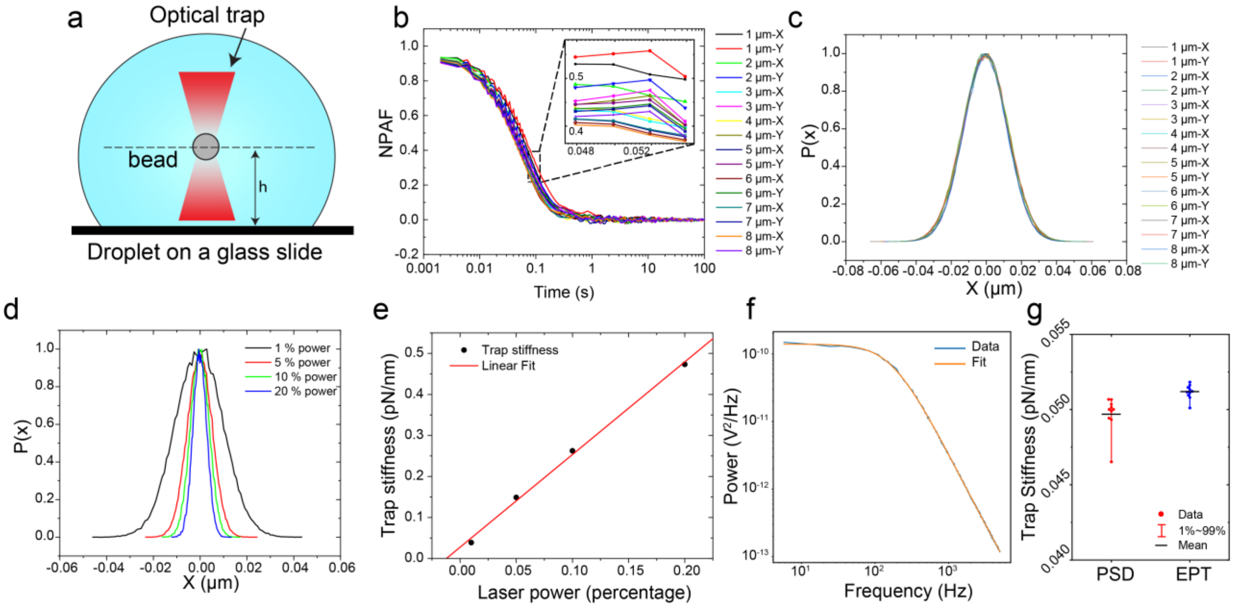

Supplementary Fig. 21. Control experiments probing the feasibility of pMOT experiments within $[KGKGG]_5$-rU40 condensates. **(a)** Scheme illustrating the optical trapping of a polystyrene microsphere within peptide-RNA droplet at distance h from the glass slide surface. **(b)** Normalized position autocorrelation function (NPAF) of a trapped microsphere within the condensate at variable distances from the glass slide surface and identical trapping power. **(c)** Probability distribution of the trapped particle displacements from the center of the optical trap at variable

distances from the glass slide surfaces. **(d)** The probability distribution of particle displacements from the center of the optical trap as a function of increasing trapping power and trapping stiffness within peptide-RNA condensate. **(e)** Trap stiffness scales linearly with laser power inside peptide-RNA condensates. **(f)** A representative power spectrum of the fluctuations of a 4.6 µm bead diffusing in water and held by an optical trap. These fluctuations are tracked through the optical trap using a quadrant photodiode (Lumicks, C-trap). The fit shown here is a Lorentzian fit ($PSD = a/(f^2 + f_c^2)$) that gives the corner frequency f_c from which the trap stiffness is calculated given the viscosity of the medium (water) is known. The PSD calibration is done through a built-in routine provided with the optical trap instrument (Lumicks, C-trap) following previously established methods[20]. **(g)** Comparison between laser-based calibration via power spectral density analysis (PSD) and the camera-based calibration via the equipartition theorem (EPT) used in this study for the same bead in (g) over multiple calibration trials. Sample size is n=10 measurements.

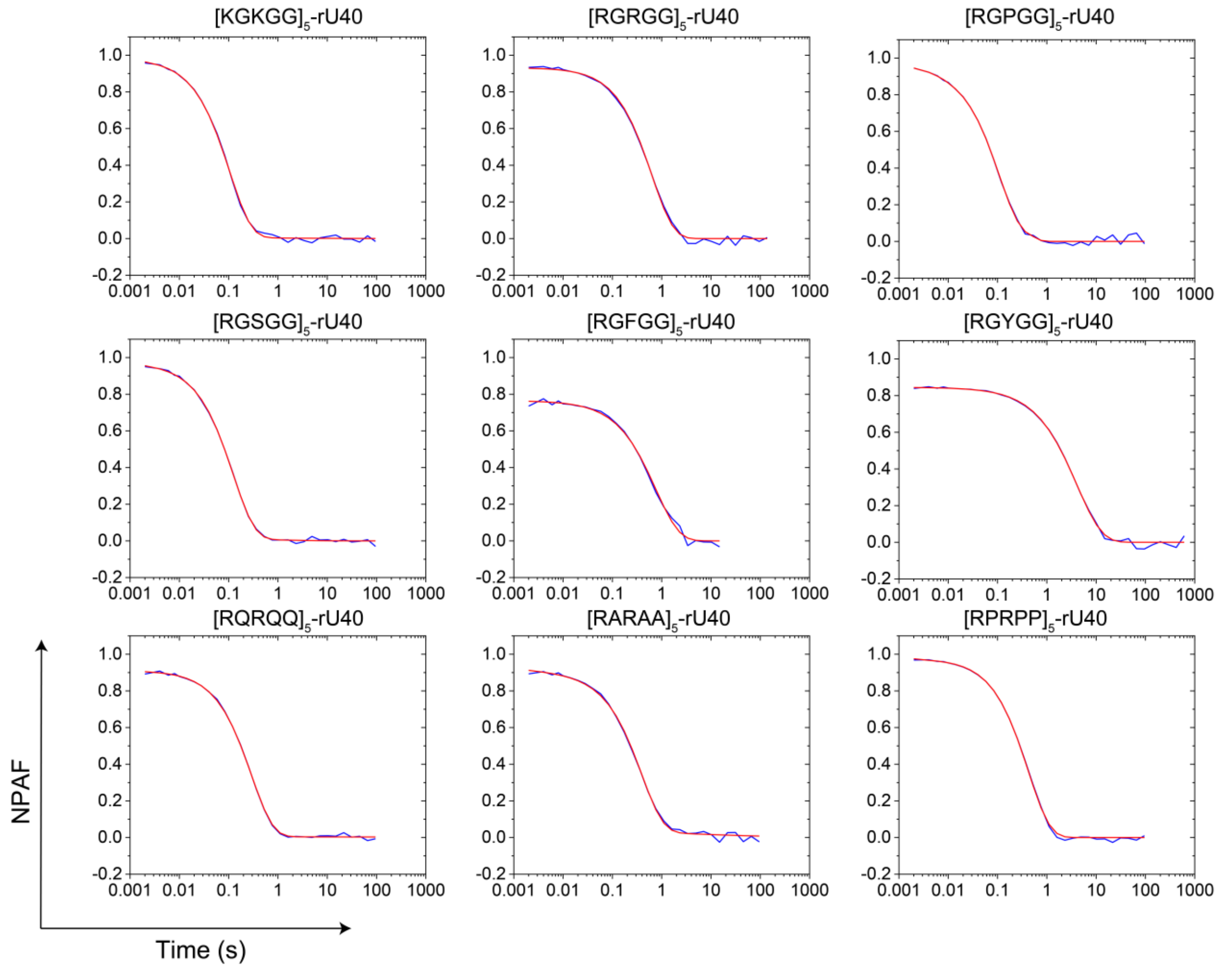

Supplementary Fig. 22. Representative plots of the calculated normalized position autocorrelation function (NPAF) and the multi-exponential fit (Equation-11) for various peptide-RNA condensate systems that are shown in Figs. 1-3 in the main text.

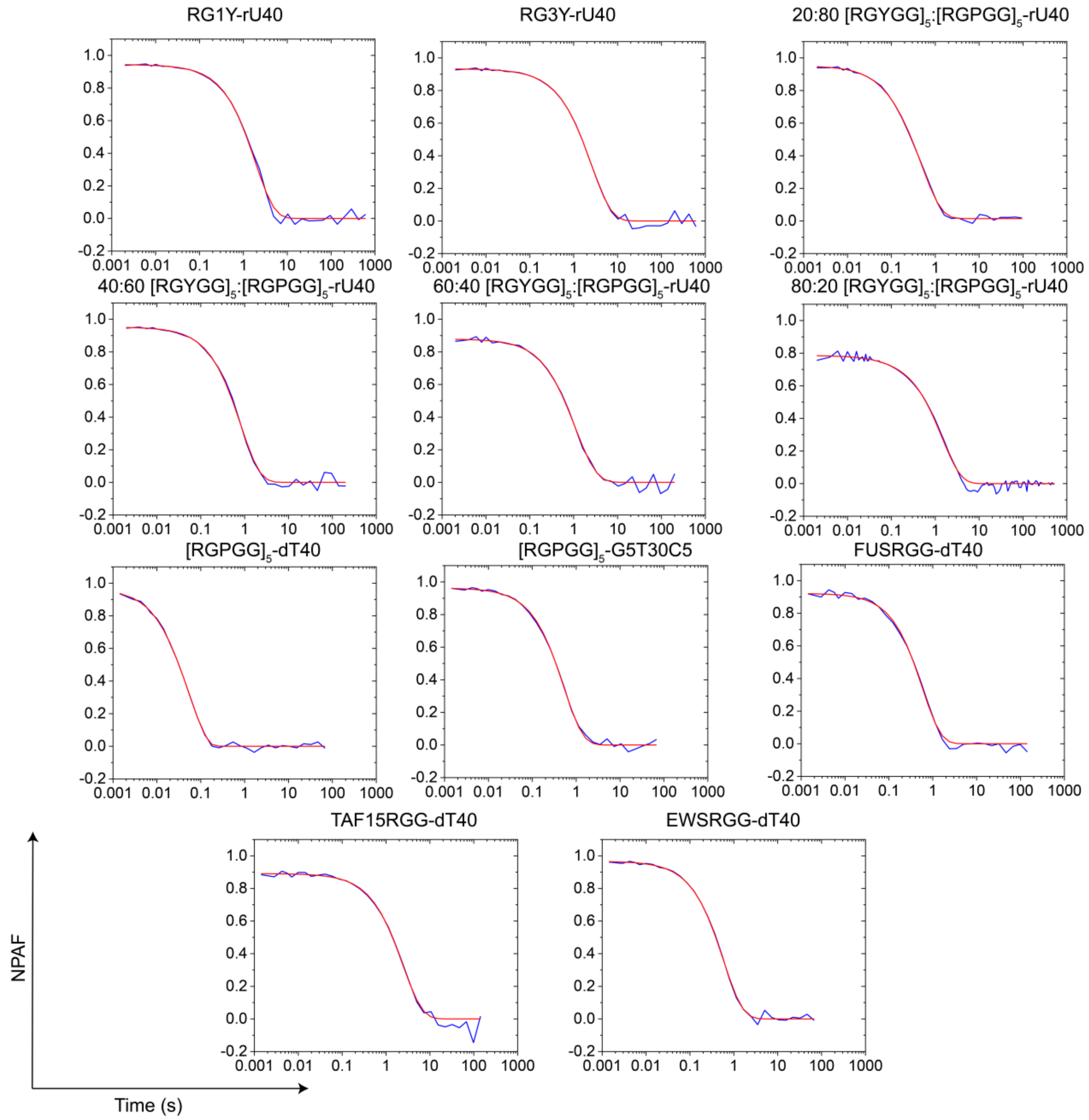

Supplementary Fig. 23. Representative plots of the calculated normalized position autocorrelation function (NPAF) and the multi-exponential fit (Equation 11) for various peptide-NA condensate systems that are shown in Figs. 5-6 in the main text and corresponding SI figures.

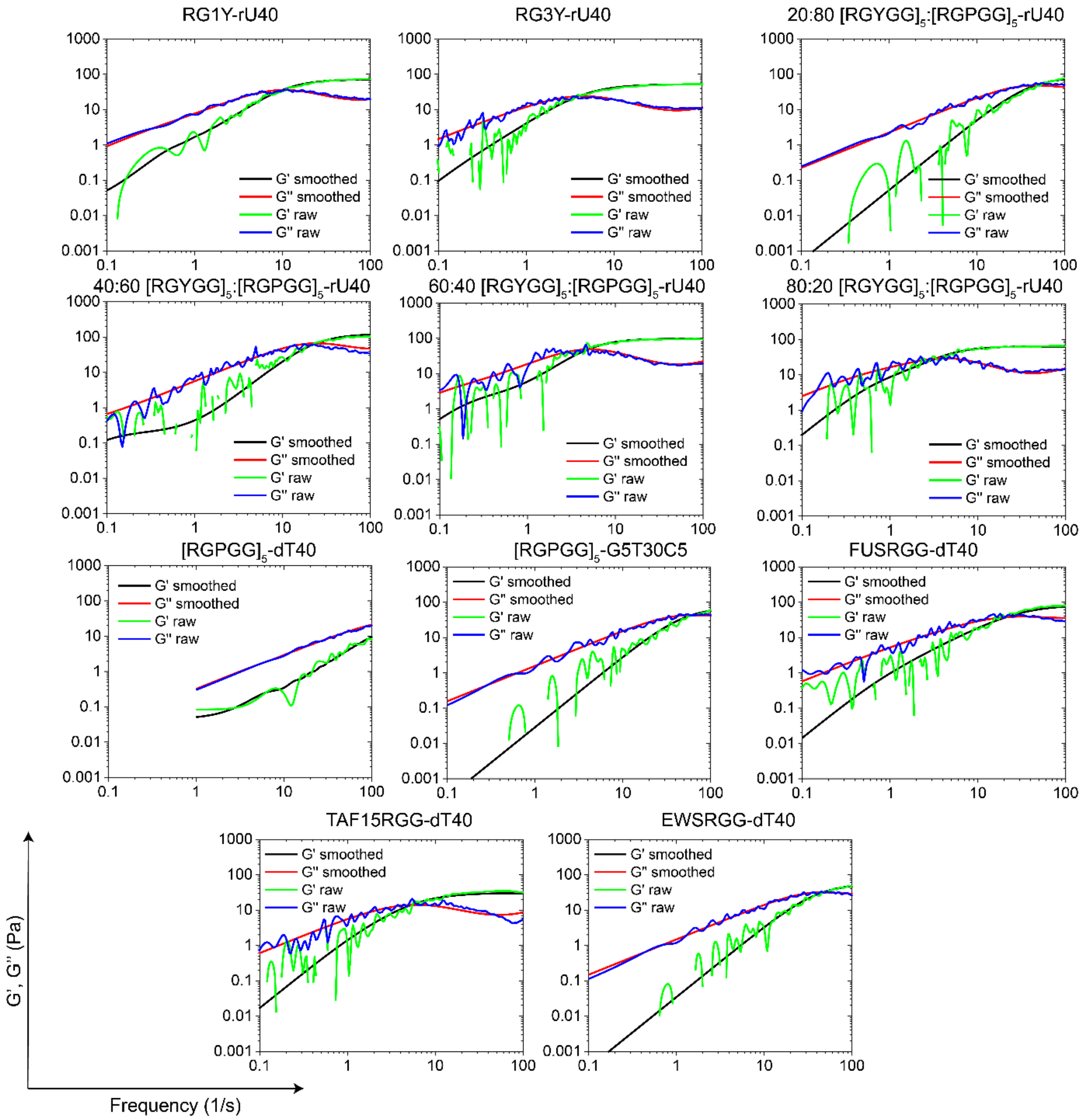

Supplementary Fig. 24. Example plots of the viscoelastic moduli calculated from raw (blue and green) and fitted (black and red) autocorrelation functions (NPAF) of various peptide-NA condensate systems shown in Figs. 5-6 in the main text and corresponding supplementary figures.

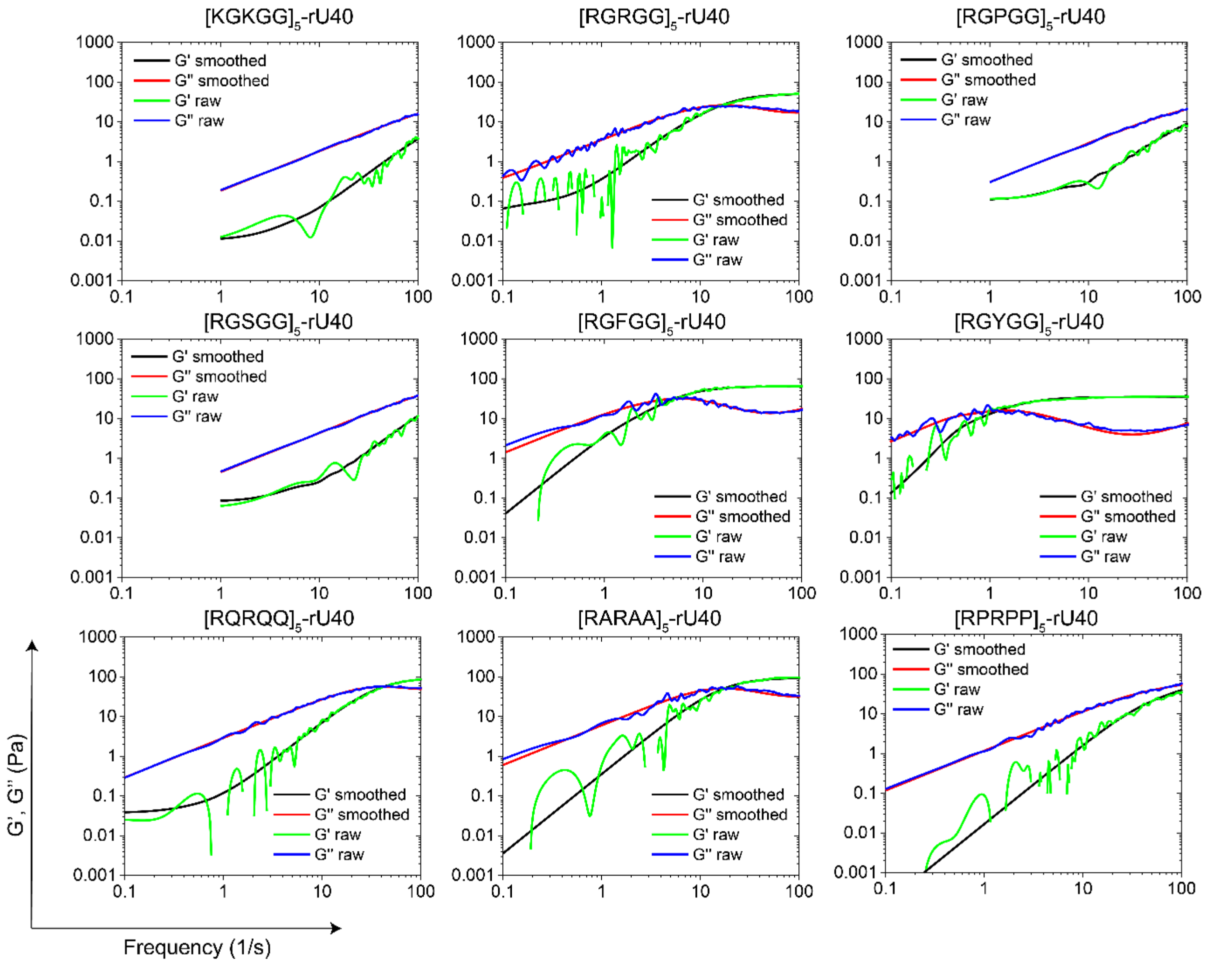

Supplementary Fig. 25. Example plots of the viscoelastic moduli calculated from raw (blue and green) and fitted (black and red) autocorrelation functions (NPAF) of various peptide-NA condensate systems that are shown in Figs. 1-3 in the main text.

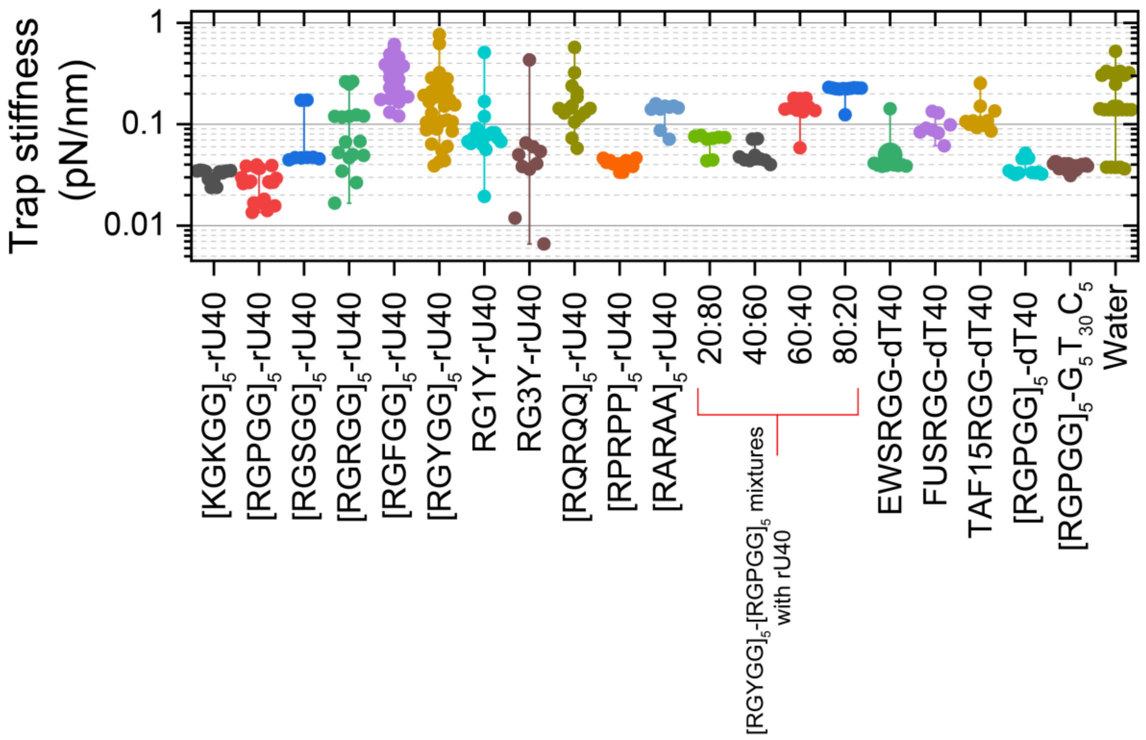

Supplementary Fig. 26. Values of the trap stiffness inside the condensate for all the condensates examined in this work as well as water. Error bars represent the range of the data. The sample size for these experiments range from 8 to 33 measurements (see the source data file for details).

Supplementary References

1 Jawerth, L. *et al.* Protein condensates as aging Maxwell fluids. *Science* **370**, 1317-1323 (2020).

2 Wei, M.-T. *et al.* Phase behaviour of disordered proteins underlying low density and high permeability of liquid organelles. *Nature chemistry* **9**, 1118-1125 (2017).

3 Good, N. E. *et al.* Hydrogen ion buffers for biological research. *biochemistry* **5**, 467-477 (1966).

4 Tassieri, M., Evans, R., Warren, R. L., Bailey, N. J. & Cooper, J. M. Microrheology with optical tweezers: data analysis. *New Journal of Physics* **14**, 115032 (2012).

5 Gieseler, J. *et al.* Optical tweezers—from calibration to applications: a tutorial. *Advances in Optics and Photonics* **13**, 74-241 (2021).

6 Neuman, K. C. & Block, S. M. Optical trapping. *Review of scientific instruments* **75**, 2787-2809 (2004).

7 Sarshar, M., Wong, W. & Anvari, B. Comparative study of methods to calibrate the stiffness of a single-beam gradient-force optical tweezers over various laser trapping powers. *Journal of biomedical optics* **19**, 115001 (2014).

8 Jun, Y., Tripathy, S. K., Narayanareddy, B. R., Mattson-Hoss, M. K. & Gross, S. P. Calibration of optical tweezers for in vivo force measurements: how do different approaches compare? *Biophysical journal* **107**, 1474-1484 (2014).

9 Mason, T. G. & Weitz, D. A. Optical measurements of frequency-dependent linear viscoelastic moduli of complex fluids. *Physical review letters* **74**, 1250 (1995).

10 Preece, D. *et al.* Optical tweezers: wideband microrheology. *Journal of optics* **13**, 044022 (2011).

11 Tassieri, M. *Microrheology with Optical Tweezers: Principles and Applications*. (CRC Press, 2016).

12 Tassieri, M. *et al.* Measuring storage and loss moduli using optical tweezers: Broadband microrheology. *Physical Review E* **81**, 026308 (2010).

13 Muller, P. Python multiple-tau algorithm (Version 0.1.9). Available at https://pypi.python.org/pypi/multipletau/ (Accessed 15 Sep 2020). (2012).

14 Evans, R., Tassieri, M., Auhl, D. & Waigh, T. A. Direct conversion of rheological compliance measurements into storage and loss moduli. *Physical Review E* **80**, 012501 (2009).

15 Huber, M. L. *et al.* New international formulation for the viscosity of H 2 O. *Journal of Physical and Chemical Reference Data* **38**, 101-125 (2009).

16 Crocker, J. C. *et al.* Two-point microrheology of inhomogeneous soft materials. *Physical Review Letters* **85**, 888 (2000).

17 Alshareedah, I., Thurston, G. M. & Banerjee, P. R. Quantifying Viscosity and Surface Tension of Multi-Component Protein-Nucleic Acid Condensates. *Biophysical journal*, doi:https://doi.org/10.1016/j.bpj.2021.01.005 (2021).

18 Hayes, L. R., Duan, L., Bowen, K., Kalab, P. & Rothstein, J. D. C9orf72 arginine-rich dipeptide repeat proteins disrupt karyopherin-mediated nuclear import. *Elife* **9**, e51685 (2020).

19 Fisher, R. S. & Elbaum-Garfinkle, S. Tunable multiphase dynamics of arginine and lysine liquid condensates. *Nature communications* **11**, 1-10 (2020).

20 Berg-Sørensen, K. & Flyvbjerg, H. Power spectrum analysis for optical tweezers. *Review of Scientific Instruments* **75**, 594-612 (2004).
