# Supplementary material for: Programmable viscoelasticity in protein-RNA condensates with disordered sticker-spacer polypeptides
Source: Nat Commun. 2021 Nov 16;12:6620. doi: 10.1038/s41467-021-26733-7 (PMC8595643; doi:10.1038/s41467-021-26733-7)
Supplement: Supplementary file 3 — Reporting Summary [file 41467_2021_26733_MOESM3_ESM.pdf]

nature portfolio

Corresponding author(s): Priya R. Banerjee

Last updated by author(s): Oct 6, 2021

Reporting Summary

Nature Portfolio wishes to improve the reproducibility of the work that we publish. This form provides structure for consistency and transparency in reporting. For further information on Nature Portfolio policies, see our Editorial Policies and the Editorial Policy Checklist.

Statistics

For all statistical analyses, confirm that the following items are present in the figure legend, table legend, main text, or Methods section.

n/a	Confirmed	
☐	☒	The exact sample size (*n*) for each experimental group/condition, given as a discrete number and unit of measurement
☐	☒	A statement on whether measurements were taken from distinct samples or whether the same sample was measured repeatedly
☒	☐	The statistical test(s) used AND whether they are one- or two-sided<br>*Only common tests should be described solely by name; describe more complex techniques in the Methods section.*
☒	☐	A description of all covariates tested
☒	☐	A description of any assumptions or corrections, such as tests of normality and adjustment for multiple comparisons
☐	☒	A full description of the statistical parameters including central tendency (e.g. means) or other basic estimates (e.g. regression coefficient) AND variation (e.g. standard deviation) or associated estimates of uncertainty (e.g. confidence intervals)
☒	☐	For null hypothesis testing, the test statistic (e.g. *F*, *t*, *r*) with confidence intervals, effect sizes, degrees of freedom and *P* value noted<br>*Give P values as exact values whenever suitable.*
☒	☐	For Bayesian analysis, information on the choice of priors and Markov chain Monte Carlo settings
☒	☐	For hierarchical and complex designs, identification of the appropriate level for tests and full reporting of outcomes
☒	☐	Estimates of effect sizes (e.g. Cohen's *d*, Pearson's *r*), indicating how they were calculated

Our web collection on statistics for biologists contains articles on many of the points above.

Software and code

Policy information about availability of computer code

Data collection	ZEN (blue, V2.3), Bluelake (v1.6.11)
Data analysis	Fiji-ImageJ13 (version 1.52p), Origin Pro (2018b), Adobe Illustrator CC (2019, v23.0). Trackmate (v6.0.1) was used for video particle tracking. Python (v3.5) was used for pMOT analysis. HOOMD-Blue 2.9.6 was used for coarse-grained molecular dynamics simulations and OpenMM. 7.5 was used for all-atom simulations. Ovito 3.5 was used for generating phase coexistence snapshots and ngvliew 3.0 was used for visualizing. all-atom residue-base interaction snapshots. All analysis and calculations were done using custom-made python (v3.5) scripts that are available on GitHub (https://github.com/l-Alshareedah/pyMOT/tree/main/Code).

For manuscripts utilizing custom algorithms or software that are central to the research but not yet described in published literature, software must be made available to editors and reviewers. We strongly encourage code deposition in a community repository (e.g. GitHub). See the Nature Portfolio guidelines for submitting code & software for further information.

Data

Policy information about availability of data

All manuscripts must include a data availability statement. This statement should provide the following information, where applicable:

- Accession codes, unique identifiers, or web links for publicly available datasets
- A description of any restrictions on data availability
- For clinical datasets or third party data, please ensure that the statement adheres to our policy

All data relevant to the findings of this manuscript are included in the manuscript, the supplementary appendix and the source data file. Additional raw data corresponding to the viscoelasticity measurements using pMOT are deposited in GitHub (https://github.com/l-Alshareedah/pyMOT/tree/main/Data).

Field-specific reporting

Please select the one below that is the best fit for your research. If you are not sure, read the appropriate sections before making your selection.

- [x] Life sciences
- [ ] Behavioural & social sciences
- [ ] Ecological, evolutionary & environmental sciences

For a reference copy of the document with all sections, see nature.com/documents/nr-reporting-summary-flat.pdf

Life sciences study design

All studies must disclose on these points even when the disclosure is negative.

Sample size	The synthetic peptides and RNA are stored as concentrated stock solutions and then diluted to final concentrations. The sample size is 5-10 micro-liters for all the experiments. This sample size was chosen because it is sufficient for microscopy analysis and cost-friendly especially that our samples are sandwiched into sealed small imaging chambers. For statistics, No calculations were done to predetermine the sample size. The sample size was chosen according to feasibility of the measurements and such that the resulting error-to-value ratio is less than 50%.
Data exclusions	No data were excluded.
Replication	All experiments reported in this manuscript have been repeated at least three times with consistent results. The viscosity measurements are repeated for at least 12 data points from different condensates across three independently prepared samples. The relaxation times are obtained from at least 12 data points from different condensates across three independent sample preparations. The VPT experiments were replicated three times across different sample preparations. Bright-field and fluorescence microscopy images are representative of a large set of images exhibiting similar features. For temperature-salt state diagrams, random points were selected and reproduced three times successfully. FRAP experiments were repeated five times (within 1-2 sample preparations) for each peptide-RNA system.
Randomization	Randomization is not relevant to this study because we are studying individual systems and not groups of systems or a specific population.
Blinding	Blinding is not relevant to this study because the experimental outcome does not depend on any decision making on part of the experimentalist, since the condensates are chosen randomly amongst a set of compositionally identical condensates belonging to a single dense phase. The appearance of all the condensates is identical. The trajectories of particles are obtained by computer-based tracking that does not need any input parameters from the user. The analysis of the results is done through a computer program and does not require any user inputs. Hence, there is no decision making throughout the experimental and analysis procedures. Therefore, blinding is not relevant.

Reporting for specific materials, systems and methods

We require information from authors about some types of materials, experimental systems and methods used in many studies. Here, indicate whether each material, system or method listed is relevant to your study. If you are not sure if a list item applies to your research, read the appropriate section before selecting a response.

Materials & experimental systems

n/a	Involved in the study	
[x]	[ ]	Antibodies
[x]	[ ]	Eukaryotic cell lines
[x]	[ ]	Palaeontology and archaeology
[x]	[ ]	Animals and other organisms
[x]	[ ]	Human research participants
[x]	[ ]	Clinical data
[x]	[ ]	Dual use research of concern

Methods

n/a	Involved in the study	
[x]	[ ]	ChIP-seq
[x]	[ ]	Flow cytometry
[x]	[ ]	MRI-based neuroimaging
